# Case Report: Bladder endometriosis presenting as a bladder mass – diagnosis and surgical management

**DOI:** 10.3389/fmed.2025.1573513

**Published:** 2025-04-30

**Authors:** Junhao Chen, Zhaojiao Li, Bo Chen, Junxian Zhao, Jieming Zuo, Haifeng Wang, Shi Fu

**Affiliations:** ^1^Department of Urology, The Second Affiliated Hospital of Kunming Medical University, Kunming, Yunnan, China; ^2^Department of First People's Hospital of Yunnan Province, Kunming, China; ^3^Department of Urology, 920th Hospital of Joint Logistics Support Force of Chinese People's Liberation Army, Kunming, Yunnan, China

**Keywords:** case report, endometriosis, deep infiltrating endometriosis, bladder endometriosis, urinary tract endometriosis

## Abstract

Endometriosis (EMs) is a common disease in women of childbearing age, categorized into ovarian, superficial, and deep infiltrating endometriosis (DIE). Bladder endometriosis (BE) is a type of DIE with an incidence of approximately 1% among women of childbearing age. Its main symptoms include pelvic discomfort and urinary dysfunction. Importantly, bladder endometriosis and ureteral endometriosis are distinct entities with different clinical manifestations and management approaches. As the condition progresses, it can involve the ureters, leading to hydronephrosis and severe renal function impairment. Currently, diagnosis primarily relies on ultrasound, MRI, and cystoscopy, with surgical treatment showing good efficacy and low recurrence rates. We report a patient with bladder endometriosis to enhance our understanding of this condition.

## Introduction

Deep infiltrating endometriosis (DIE) is the most severe type of EM, estimated to affect approximately 1% of women of childbearing age. Approximately 1% of endometriosis patients have urinary tract endometriosis (UTE), with bladder involvement being the most common, accounting for 70–85% (1). It is essential to note that bladder endometriosis and ureteral endometriosis are separate conditions, each with distinct pathophysiological mechanisms and clinical presentations. While bladder endometriosis primarily affects the bladder wall, ureteral endometriosis involves the ureter, potentially leading to obstructive uropathy and renal impairment. It typically progresses from the serosal surface of the bladder toward the mucosa, often presenting as multifocal lesions, with the trigone and the dome of the bladder being the most commonly affected areas (2, 3). The implantation theory and the origin theory are currently better explanations for BE. Notably, endometriosis, including BE, is a multidisciplinary condition requiring collaboration among urologists, gynecologists, radiologists, and other specialists for effective diagnosis and management. Given its frequent involvement of the urinary tract, bladder endometriosis underscores the critical role of urologists in its diagnosis and management, collaborating with gynecologists to address its multidisciplinary nature. Most patients experience discomfort during their menstrual cycle, which overlaps with the discomfort caused by normal menstruation, making the diagnosis of BE challenging. Therefore, discomfort experienced during a patient’s menstrual cycle should not be solely attributed to gynecological issues.

## Case report

**Figure 1:**
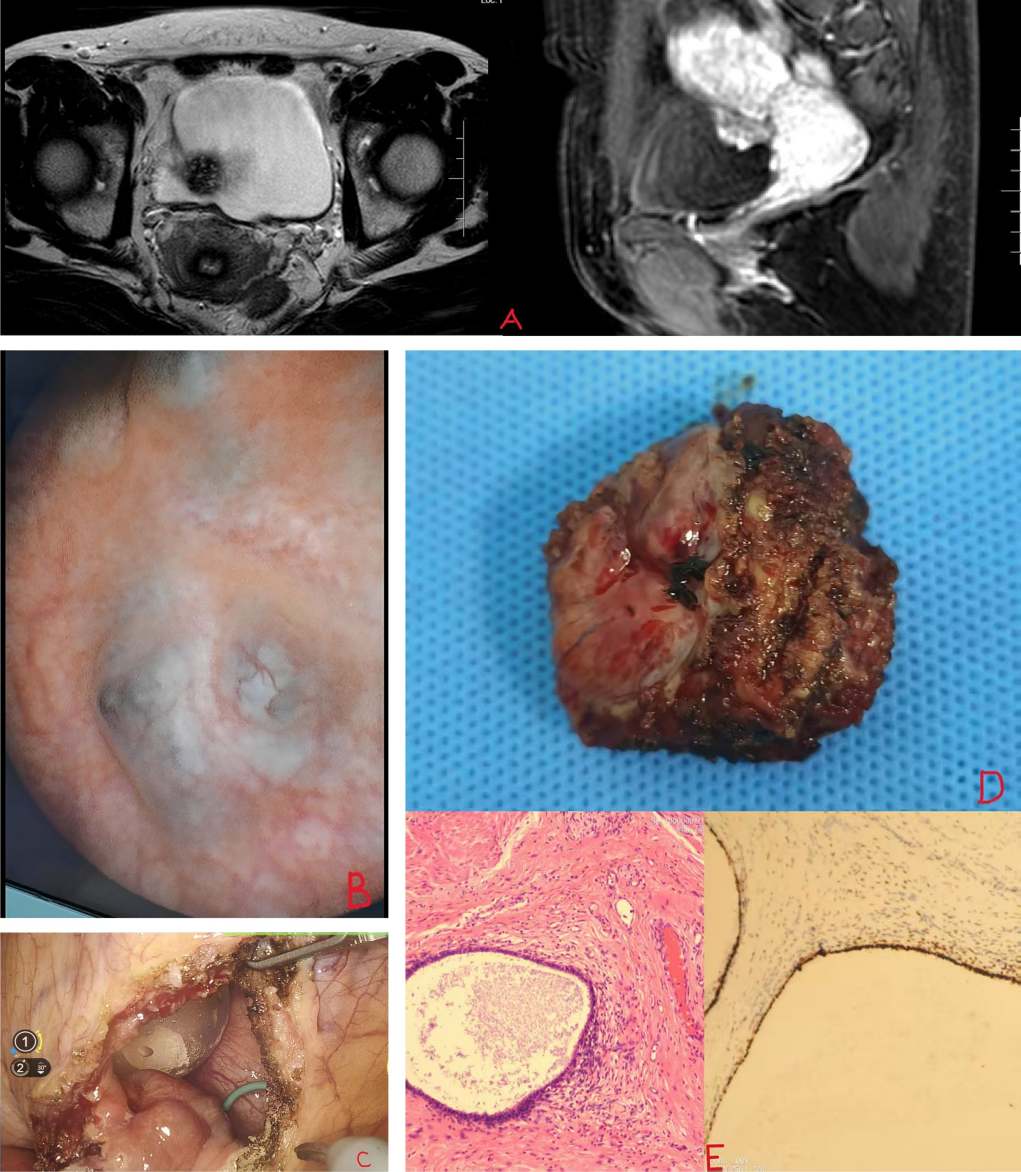
**(A)** MRI shows bladder mass. **(B,C)** Blue-purple nodules at the base of the bladder. **(D,E)** Bladder endometriosis and PR(+).

The patient is a 40-year-old female who was admitted to the hospital due to intermittent lower abdominal discomfort over the past year. She has no hematuria, no urinary frequency, no urgency, no loin ache, back pain, no history of urinary retention, no abnormal vaginal bleeding, and no dyspareunia. The patient thought it was caused by the normal menstrual cycle and did not undergo any special treatment. One week ago, during a gynecological transabdominal ultrasound, a bladder-occupying lesion was discovered, measuring approximately 2*3 cm. This mass needs to be differentiated from bladder leiomyoma and bladder cancer. Previously in good health, but it is worth noting that she has had three previous cesarean sections. After admission, further pelvic MRI revealed a mass on the right posterior wall of the bladder, measuring approximately 2.0*2.0*1.8 cm, with no significant enhancement on contrast (Figure 1A). CT scan indicated a bladder mass without apparent hydronephrosis. Urine methylated is negative, Cr: 69 μmol/L, and there are no abnormalities in the routine urine examination. Subsequently, the patient underwent cystoscopy, during which a blue-red nodular protrusion was observed on the posterior wall of the bladder, growing from the outside inward, without the typical characteristics of urothelial carcinoma. A biopsy was taken, and the pathological examination results indicated a tendency toward endometriosis. One week later, the patient underwent a procedure involving the implantation of a right ureteral stent and a robot-assisted laparoscopic partial cystectomy. After anesthesia, the patient was placed in the lithotomy position, and a standard ureteroscope was used to re-evaluate the lesion. The mass was located on the right posterior wall of the bladder, approximately 3 cm from the right ureteral orifice and 4 cm from the left ureteral orifice. To prevent ureteral injury, 5F double-J stents were placed bilaterally under the guidance of a Cook single soft guidewire. The patient was then repositioned in a supine V-shaped position. A supraumbilical observation port was established, and under laparoscopic visualization, puncture ports were placed 3 cm left of the umbilicus, three transverse fingers above the umbilicus on the left, 4 cm right of the umbilicus, and at the intersection of the right anterior axillary line and the line connecting the bilateral iliac spines. These ports were connected to the da Vinci Xi robotic system. After freeing the colon, a mass approximately 4 cm in size was identified on the posterior bladder wall. The peritoneum above the uterovesical space was incised with an Endoknife, exposing the bladder. The bladder serosa, fat, and muscle layers were sequentially opened, revealing urine leakage into the abdominal cavity. The bladder wall was incised outward from the base, and the mass was circumferentially dissected clockwise, removed, and placed in a specimen bag. The bladder wall was sutured using 2–0 Ethicon absorbable sutures (SXPP1B410 2–0) in two layers: the first layer included the muscle and mucosa, and the second reinforced the serosa to the muscle. Water injection through the urinary catheter confirmed no leakage, indicating a secure closure. A Xin Sanrui abdominal drainage tube was placed, and the specimen was retrieved. Intraoperative blood loss was approximately 50 mL, and the procedure lasted 140 min (Figures 1B,C). Postoperative pathological examination indicated endometriosis. Immunohistochemistry results showed: PR(+), ER(+), VIM(+), Ki-67(3%), P40(−), PAX-8(+), GATA3(−), Villin(−), CK20(−), and CX7(+) (Figures 1D,E). No hormone therapy was used postoperatively. Postoperative abdominal drainage output decreased from 40 mL to 10 mL to 0 mL, and the drainage tube was removed on the third day. The urinary catheter was retained for 2 weeks, with follow-up urinalysis showing no significant urinary tract infection, hematuria, frequency, urgency, or dysuria, and voiding was normal after catheter removal. One month later, upon reexamination, there was no hydronephrosis in the kidneys. The ureteral stent was removed, and cystoscopy revealed no nodules with no signs of recurrence. The lower abdominal discomfort during menstruation has notably improved compared to before.

## Discussion

The BE can be divided into primary and secondary types. Primary BE has two main theories: one suggests that menstrual endometrium refluxed through the urethra implants on the peritoneal surface, while the other proposes that BE results from the extension of Müllerian remnants’ metaplasia and/or glandular smooth muscle proliferation, as its histological features consist of dense tissues composed of fibers and smooth muscle cells with islands or clusters of glands and stroma (4). It is crucial to differentiate bladder endometriosis from ureteral endometriosis, as the latter often presents with hydronephrosis and renal impairment due to ureteral obstruction, requiring distinct diagnostic and therapeutic approaches. In our case, beyond histopathological confirmation of endometriosis, immunohistochemistry (IHC) played a significant role in characterizing the lesion. The positive staining for progesterone receptor (PR), estrogen receptor (ER), and vimentin (VIM), alongside negative markers such as P40, GATA3, and CK20, supported the diagnosis of endometriosis by highlighting its hormonal dependency and mesenchymal origin, while excluding epithelial malignancies such as urothelial carcinoma. The low Ki-67 index (3%) further indicated a benign proliferative behavior, aligning with the clinical outcome of no recurrence post-surgery. IHC is particularly valuable in such cases, as it enhances diagnostic specificity when bladder lesions mimic malignancies, guiding surgical planning and postoperative management by confirming the endometriotic nature of the tissue. In this case, the involvement of a urologist was pivotal, as cystoscopy not only facilitated the identification of the bladder lesion but also allowed for a precise biopsy, distinguishing BE from other bladder pathologies such as leiomyoma or carcinoma. Moreover, the use of a ureteral stent and robotic-assisted laparoscopic partial cystectomy highlights the urological expertise required to preserve bladder integrity and prevent urinary complications. Furthermore, in the evaluation of patients with BE, it is essential to exclude the presence of concomitant adenomyosis, especially in those presenting with dysmenorrhea and abnormal uterine bleeding (AUB). Adenomyosis, characterized by the presence of endometrial tissue within the myometrium, frequently coexists with endometriosis and may exacerbate symptoms such as pelvic pain and menstrual irregularities. Recent studies suggest that patients with both BE and adenomyosis may experience more severe and persistent symptoms, even after BE treatment, due to the independent pathological effects of adenomyosis on the uterine structure and function (5). Moreover, it is important to note that even mild symptoms in the presence of adenomyosis may persist. This is because adenomyosis can cause chronic inflammation and structural changes within the uterine wall, leading to ongoing pelvic discomfort and menstrual irregularities. Recent research has highlighted that adenomyosis-related symptoms, such as dysmenorrhea and AUB, may not fully resolve with treatments targeting endometriosis alone, emphasizing the need for a comprehensive approach to address both conditions (6). Beyond diagnosis, urologists play a vital role in surgical management, leveraging their knowledge of pelvic anatomy and reconstructive techniques to minimize complications such as recurrence or voiding dysfunction, which are notably higher with transurethral resection approaches compared to laparoscopy. The latter is often associated with iatrogenic lesions such as pelvic surgery, suggesting that it results from the dispersion of endometrial cells during surgery and damage to the uterine incision. This highlights the importance, especially during cesarean section procedures, of preventing the intraoperative implantation of endometrial cells in other locations (7). Approximately 30% of patients with BE are asymptomatic, and the diagnosis is incidental. In fact, its typical presentation includes symptoms such as urinary frequency, urgency, burning sensation, dysuria, urinary retention, discomfort and pain above the pubic bone, and acute urethral syndrome (8).

Currently, the diagnosis of BE mainly relies on transvaginal ultrasound (TVS), pelvic magnetic resonance imaging (MRI), and cystoscopy. An abdominal ultrasound has lower accuracy than TVS due to interference from the abdominal wall and intestines. Transvaginal ultrasound has an overall sensitivity and specificity of 62 and 100%, respectively, for detecting bladder endometriosis (9). There are reports of using three-dimensional pelvic ultrasound to diagnose bladder endometriosis. Color Doppler can assess the distance between the ureter and the lesion and perform virtual cystoscopy. The sensitivity is 25%, and the specificity is 100% (10). While the corresponding percentages reported by Exacoustos et al. (11) were 100 and 96.8%, respectively. There may be a correlation with the experience of the sonographer. Some studies suggest that adding three-dimensional reconstruction does not improve the performance of TVS in diagnosing BE, but three-dimensional ultrasound can more accurately assess the volume of BE (12). MRI has a high sensitivity of up to 88% and a high specificity of 99% in diagnosing BE, with an accuracy of approximately 98%, demonstrating good diagnostic performance (13). Some scholars have evaluated the distance between BE lesions diagnosed by MRI and the ureteral orifice, showing good accuracy for lesions in the cul-de-sac, pouch, and bladder base, all exceeding 95%. This can better predict the need for ureteral resection-reimplantation (14). As an invasive procedure, the greatest advantage of cystoscopy is the ability to perform biopsies for pathological verification, determine the location and extent of lesions, and provide precise assessment for subsequent surgeries. However, in reality, during menstruation, endometriotic nodules in the bladder are larger, more congested, and appear clearer under cystoscopy, which also poses limitations to the procedure. Research has reported that dynamic cystoscopy appears to be a highly specific examination (97.78%) but with lower sensitivity (58.21%), showing better judgment for submucosal lesions (15). Treatment for BE is mainly divided into conservative and surgical treatments. The former relies primarily on medications such as gonadotropin-releasing hormone (GnRH) agonists and antagonists, progestins, and combined oral contraceptives, which have shown good efficacy. These medications are often used as the initial treatment method for young women and women who wish to preserve fertility (16, 17). However, surgical treatments such as partial cystectomy are considered effective methods for the complete removal of BE lesions and ensuring long-term relief (18). Research using CO laparoscopy to remove BE shows a recurrence rate of 3.4%. Among 94 previously infertile patients, 74.5% achieved pregnancy, with 50% conceiving spontaneously (19). Some scholars have used cystoscopy in combination with laparoscopy during surgery to remove lesions, a technique known as the STT (see-through technique), which can accurately identify the edges of the affected area and the true incision line. In all cases, the margins were negative, with an average follow-up of 20.4 months showing no recurrence (20). The classic use of transurethral resection of bladder tumor (TURBT) for bladder masses is not suitable for BE. Due to the nodules developing from the outer to the inner layers of the bladder wall, complete removal of endometriotic lesions is nearly impossible. This puts patients at a high risk of bladder perforation, short-term symptoms, and disease recurrence, making this treatment option generally unsuitable (21).

## Conclusion

The BE is relatively rare, and its diagnosis primarily relies on TVS, pelvic MRI, and cystoscopy, each with its own advantages and limitations. Pelvic MRI has high diagnostic accuracy and good patient acceptance, making it a necessary examination. Surgical treatment can greatly relieve patient symptoms with good outcomes. While current medications have decent efficacy, long-term use of hormonal drugs may potentially impact overall health, making them suitable as the initial treatment method for young women and those wishing to preserve fertility. Additionally, healthcare providers should develop personalized diagnosis and treatment plans based on each patient’s specific condition. Collaborating with gynecologists and radiologists is crucial for effective treatment (17).

## Data Availability

The original contributions presented in the study are included in the article/supplementary material, further inquiries can be directed to the corresponding authors.
